# Diagnosis and Management of Contemporary Issues in Maternal-Fetal Medicine

**DOI:** 10.3390/diagnostics16111633

**Published:** 2026-05-27

**Authors:** Antonios Siargkas, Panagiota Kripouri, Themistoklis Dagklis, Ioannis Tsakiridis

**Affiliations:** Third Department of Obstetrics and Gynecology, School of Medicine, Faculty of Health Sciences, Aristotle University of Thessaloniki, 54124 Thessaloniki, Greecetdagklis@yahoo.gr (T.D.)

## 1. Introduction

Pregnancy is a profound physiological stage in the cycle of life, yet it remains a complex diagnostic puzzle for medical specialties outside of obstetrics. This challenge is growing increasingly acute today: shifting demographic trends—such as advanced maternal age, rising obesity rates, and the increased prevalence of pre-existing maternal comorbidities—have led to a surge in high-risk pregnancies globally [1]. Consequently, maternal mortality and severe morbidity rates have seen concerning increases, driven largely by cardiovascular conditions, sepsis, and delayed acute care [1,2].

The historical perception that pregnant patients are exceptionally fragile, coupled with the limitations that pregnancy imposes on the use of radiological imaging, frequently leads clinicians outside of obstetrics to adopt a defensive and hesitant stance. This fosters a dangerous diagnostic bias wherein emerging, potentially life-threatening symptoms are erroneously attributed to the physiological adaptations of pregnancy alone. This phenomenon is further compounded by the absence of a globally standardized, cross-specialty curriculum in obstetrics [3], a vulnerability recently exacerbated by the COVID-19 pandemic, which restricted hands-on training opportunities for medical trainees [4].

The consequences of these systemic gaps are tangible. Recent reports emphasize that the vast majority of pregnancy-related deaths remain preventable, with fragmented care, diagnostic delays, and poor cross-specialty communication identified as critical contributing factors [2]. In response, public health initiatives such as the “Hear Her” campaign have aimed to sensitize healthcare providers and raise awareness, showing promising early results in mitigating maternal morbidity [5]. However, as the clinical complexity of maternal care deepens, isolated obstetric practice is no longer sufficient; a whole-system, multidisciplinary approach—integrating cardiologists, surgeons, and maternal–fetal medicine specialists—is essential to optimize patient safety.

This Special Issue, “Diagnosis and Management of Contemporary Issues in Maternal-Fetal Medicine,” serves as an academic extension of these clinical and advocacy efforts. By investigating overlooked maternal conditions, acute surgical emergencies, and the intricate physiology of the fetoplacental unit, this collection aims to bridge the gap between obstetric and general medical care, fostering a truly multidisciplinary approach to perinatal health.

## 2. Overview of the Published Papers

The seven papers featured in this Special Issue address a broad spectrum of clinical challenges, ranging from acute maternal emergencies to nuanced fetal diagnostics and complex ethical dilemmas.

### 2.1. Maternal Medical and Surgical Emergencies

Acute conditions during pregnancy require high clinical suspicion and swift, coordinated action. Didžiokaitė et al. (contribution 1) present a compelling case report regarding the delayed diagnosis of acute appendicitis in a third-trimester patient. Because the physiological changes of pregnancy often obscure typical clinical presentations, the patient suffered severe postoperative complications. The authors highlight these diagnostic pitfalls and report the rare but successful application of vacuum-assisted closure (VAC) therapy during pregnancy, emphasizing the necessity of seamless cross-specialty collaboration.

Similarly, Ştefan et al. (contribution 2) explore the complex management of severe aortic stenosis on a bicuspid valve coupled with pre-excitation syndrome in a pregnant patient. This case underscores the profound cardiovascular demands of pregnancy that can unmask high-risk maternal cardiac conditions. The authors detail a successful multidisciplinary approach to managing subsequent fetal growth restriction and postpartum complications, proving that strict monitoring by a joint cardio-obstetric team is paramount.

### 2.2. Placental Pathology and Perinatal Infections

The placenta’s vital role as a mediator of fetal health is a major focus of this Issue. Gerede et al. (contribution 3) provide a comprehensive systematic review of the “placenta–heart axis” in congenital heart disease (CHD). By synthesizing data from modern imaging modalities, they demonstrate that fetuses with CHD frequently exhibit altered placental vascularization, reduced volumetric growth, and impaired oxygenation. This suggests that the placenta may actively contribute to CHD pathophysiology rather than act as a passive bystander.

Further investigating placental biomarkers, Siargkas et al. (contribution 4) contribute a systematic review and meta-analysis on premature placental calcification. Their findings re-establish the clinical utility of ultrasound placental grading, proving that grade 3 calcification before 36 weeks of gestation is significantly associated with adverse outcomes, including small-for-gestational-age (SGA) neonates, preeclampsia, and fetal growth restriction.

Infections involving the fetoplacental unit are critically examined by Babacheva et al. (contribution 5). In their retrospective cohort study, the authors investigated the association between maternal chorioamnionitis and neonatal sepsis in preterm infants born before 32 weeks of gestation. The research reveals that chorioamnionitis is a strong, independent predictor of early-onset neonatal sepsis (EOS), highlighting the critical need for targeted infectious surveillance in this highly vulnerable population.

### 2.3. Fetal Growth and Complex Interventions

Redefining traditional thresholds for fetal well-being is vital for modern obstetric care. Mendez-Piña et al. (contribution 6) challenge the conventional dichotomization of fetal growth in a retrospective cohort study. They found that fetuses with an estimated fetal weight (EFW) between the 10th and 20th percentiles at 28–30 weeks still carry a significantly increased risk of neonatal morbidity, progression to overt growth restriction, and low birth weight when compared to those in the 20th–90th percentiles.

Finally, navigating high-risk pregnancies inevitably leads to profound ethical and clinical dilemmas. Dinu et al. (contribution 7) offer a comprehensive review of the therapeutic termination of pregnancy. By synthesizing current knowledge on diagnostic screening methods, genetic determinants, and environmental factors, the authors shed light on the heavy psychopathological and socio-economic burdens influencing these decisions. They stress the critical importance of empathetic, multidisciplinary counseling for women facing these traumatic obstetric events.

## 3. Conclusions

The research and clinical insights compiled in this Special Issue illuminate the intricacies of modern maternal–fetal medicine. Whether re-evaluating fetal growth percentiles, pioneering placental imaging, or managing acute maternal emergencies, the findings herein reinforce a singular truth: optimal pregnancy care cannot exist in a silo. We extend our deepest gratitude to all the contributing authors and dedicated reviewers. We hope this Special Issue serves as a valuable resource for obstetricians, maternal–fetal medicine specialists, and general practitioners, ultimately guiding earlier interventions and improving outcomes for both mother and child.
